# Anesthesia Clinical Workload Estimated From Electronic Health Record Documentation vs Billed Relative Value Units

**DOI:** 10.1001/jamanetworkopen.2023.28514

**Published:** 2023-08-11

**Authors:** Sunny S. Lou, Laura R. Baratta, Daphne Lew, Derek Harford, Michael S. Avidan, Thomas Kannampallil

**Affiliations:** 1 Department of Anesthesiology, Washington University School of Medicine, St Louis, Missouri; 2 Institute for Informatics, Washington University School of Medicine, St Louis, Missouri; 3 Division of Biostatistics, Washington University School of Medicine, St Louis, Missouri

## Abstract

**Importance:**

Accurate measurements of clinical workload are needed to inform health care policy. Existing methods for measuring clinical workload rely on surveys or time-motion studies, which are labor-intensive to collect and subject to biases.

**Objective:**

To compare anesthesia clinical workload estimated from electronic health record (EHR) audit log data vs billed relative value units.

**Design, Setting, and Participants:**

This cross-sectional study of anesthetic encounters occurring between August 26, 2019, and February 9, 2020, used data from 8 academic hospitals, community hospitals, and surgical centers across Missouri and Illinois. Clinicians who provided anesthetic services for at least 1 surgical encounter were included. Data were analyzed from January 2022 to January 2023.

**Exposure:**

Anesthetic encounters associated with a surgical procedure were included. Encounters associated with labor analgesia and endoscopy were excluded.

**Main Outcomes and Measures:**

For each encounter, EHR-derived clinical workload was estimated as the sum of all EHR actions recorded in the audit log by anesthesia clinicians who provided care. Billing-derived clinical workload was measured as the total number of units billed for the encounter. A linear mixed-effects model was used to estimate the relative contribution of patient complexity (American Society of Anesthesiology [ASA] physical status modifier), procedure complexity (ASA base unit value for the procedure), and anesthetic duration (time units) to EHR-derived and billing-derived workload. The resulting β coefficients were interpreted as the expected effect of a 1-unit change in each independent variable on the standardized workload outcome. The analysis plan was developed after the data were obtained.

**Results:**

A total of 405 clinicians who provided anesthesia for 31 688 encounters were included in the study. A total of 8 288 132 audit log actions corresponding to 39 131 hours of EHR use were used to measure EHR-derived workload. The contributions of patient complexity, procedural complexity, and anesthesia duration to EHR-derived workload differed significantly from their contributions to billing-derived workload. The contribution of patient complexity toward EHR-derived workload (β = 0.162; 95% CI, 0.153-0.171) was more than 50% greater than its contribution toward billing-derived workload (β = 0.106; 95% CI, 0.097-0.116; *P* < .001). In contrast, the contribution of procedure complexity toward EHR-derived workload (β = 0.033; 95% CI, 0.031-0.035) was approximately one-third its contribution toward billing-derived workload (β = 0.106; 95% CI, 0.104-0.108; *P* < .001).

**Conclusions and Relevance:**

In this cross-sectional study of 8 hospitals, reimbursement for anesthesiology services overcompensated for procedural complexity and undercompensated for patient complexity. This method for measuring clinical workload could be used to improve reimbursement valuations for anesthesia and other specialties.

## Key Points

**Question:**

How does reimbursement in anesthesiology compare with an electronic health record (EHR)—derived measure of clinical workload?

**Findings:**

In this cross-sectional study of 31 688 anesthetic encounters across 8 hospitals, reimbursement appeared to overvalue procedural complexity by 3-fold and undervalue patient complexity by one-third compared with EHR-derived workload.

**Meaning:**

These findings suggest that anesthesia reimbursement may not adequately reflect the cognitive and physical effort of providing anesthesiology services, especially for patients with more severe illness.

## Teaser

This cross-sectional study compares anesthesia clinical workload estimated from electronic health record (EHR) audit log data vs billed relative value units.

## Introduction

Accurate measurement of clinical workload can inform many aspects of health care policy. For example, increased workload is associated with increased risk of burnout,^1,2,3^ and organizational platforms to monitor and streamline clinical work may help improve clinician wellness.^4,5^ In addition, measurement of clinical workload is instrumental in designing health care reimbursement. For example, the US Centers for Medicare & Medicaid Services (CMS) devised the relative value unit (RVU) fee schedule to capture the time and intensity (ie, effort, skill, and stress) for specific clinical services.^6^ However, CMS relies largely on physician survey data to determine time and intensity, which has been criticized for inaccuracy, infrequent adjustments for changes in clinical practice, and potential conflicts of interest.^7,8,9,10,11^ There is a need for more quantitative, scalable, and unbiased methods for measuring clinical workload to better guide health care policy.

Most traditional methods for measuring clinical workload have relied on either survey data or direct observation of clinical activities.^12^ Such methods are limited because they are labor-intensive and subject to confounding due to sampling and recall bias or Hawthorne effects; therefore, obtaining reliable workload measures across large clinician populations has been challenging. In this study, we had the following research aims: (1) to develop an objective and scalable approach for measuring clinical workload using electronic health record (EHR) audit logs and (2) to compare this EHR-derived workload metric with a standard measure of workload, namely the billed RVUs.

## Methods

### Study Setting, Design, and Participants

This was a cross-sectional study conducted at 8 hospitals and outpatient surgical centers affiliated with BJC Healthcare and the Washington University School of Medicine. These hospitals encompass both academic and community practice settings and serve a diverse rural, suburban, and urban population across 2 states. This study included all anesthesiology clinicians (eg, attending anesthesiologists, certified registered nurse anesthetists, trainee physicians and nurses) who provided direct anesthetic care at one of the study hospitals between August 26, 2019, and February 9, 2020. The study protocol was approved by the Institutional Review Board of Washington University with a waiver of informed consent, as the study involved minimal risk to participants and could not be conducted without the waiver. This study is reported according to the Strengthening the Reporting of Observational Studies in Epidemiology (STROBE) reporting guideline.^13^

### Measuring EHR-Derived Workload

Raw audit log data on EHR (Epic Systems) use was extracted from the ACCESS_LOG table in Epic’s Clarity database for all anesthesiology clinicians who attested to providing direct anesthetic care for at least 1 surgical encounter during the study period. Audit logs capture clinician click actions within the EHR that result in the display or modification of patient data and are mandated for security purposes by the Health Insurance Portability and Accountability Act. Each click action generates a record of what action was performed, time stamp of the action, patient on which the action occurred, and user responsible for the action (eFigure 1 in Supplement 1); because most clinical work is documented in the EHR, audit logs have previously been used to measure clinical workflow and workload.^3,14,15,16^

To summarize EHR activity at the level of the anesthetic encounter, audit logs were aggregated across all clinicians attesting to providing anesthetic care for each encounter and subdivided into the preoperative, intraoperative, and postoperative phases of care based on timestamp relative to the anesthesia start and stop times. Because encounter-level identifiers were not reliably present in the audit log data, patient identifiers were used to link audit log actions to anesthetic encounters.

Patients with multiple encounters within a month were excluded due to ambiguity in assigning audit log actions to a specific encounter. Encounters were also excluded if the American Society of Anesthesiology (ASA) physical status score was 6, if the procedural service was gastroenterology (due to the high volume and rapid turnover of these procedures resulting in different EHR use patterns), if no billing information was available, or if the encounter was reimbursed using alternative billing mechanisms (eg, labor epidurals, cosmetic surgery) (eFigure 2 in Supplement 1).

For each encounter, the following outcomes were measured: total count of EHR-based actions observed and the count of actions subdivided across the preoperative, intraoperative, and postoperative phases of care. Given that meaningful anesthesia care occurred throughout the preoperative, intraoperative, and postoperative period, EHR-derived workload was conceptualized as the total count of all audit log actions performed by all members of the anesthesia care team throughout the perioperative period. We also measured the timing of preoperative actions relative to anesthesia start, intraoperative actions relative to anesthesia start and stop (ie, normalized so 0 is anesthesia start and 1 is anesthesia stop), and postoperative actions relative to anesthesia stop.

### Measuring Patient Complexity, Procedural Complexity, Anesthesia Duration, and Billing-Derived Workload

The ASA has devised standardized methods for scoring patient and procedural complexity to facilitate billing for anesthesia services. The ASA measures patient complexity using the ASA physical status score, which ranges from 1 to 5, with 1 being a healthy patient with no medical problems and 5 being a moribund patient.^17^ A patient’s ASA physical status is scored on the day of surgery by the supervising anesthesiologist. The ASA modifier maps the ASA physical status score to billable units according to the following scale: ASA physical status of 1 and 2 are mapped to an ASA modifier of 0; ASA physical status of 3 to an ASA modifier of 1; ASA physical status of 4 to an ASA modifier of 2; and ASA physical status of 5 to an ASA modifier of 3.

The ASA defines procedural complexity using a base unit value. Each anesthesia *Current Procedural Terminology* (*CPT*) code has a corresponding base unit value reflecting procedural complexity; these values are assigned by expert consensus and range from 3 to 20.^18,19^ Anesthesia *CPT* codes are determined based on the primary surgical *CPT* code for the performed procedure.

Payments for anesthesia services in the US are determined by the ASA Relative Value Guide.^18^ For each encounter, total billed units equal the sum of the patient’s ASA modifier, the procedural base unit value, and anesthesia time units, where one time unit is awarded for every 15 minutes of anesthesia duration. Payments are then equal to the total billed units multiplied by a conversion factor depending on insurance coverage.

For this study, billing information was extracted for all anesthetic encounters from the departmental billing database, including the assigned ASA physical status score and modifier, the procedural base unit value, anesthesia duration and time units, and total billed units. For the statistical model, ASA modifier was used as the measure of patient complexity, the procedural base unit value was used as the measure of procedure complexity, and time units was used as the measure of anesthesia duration. Total billed units were used as the measure of billing-derived workload.

### Statistical Analysis

Descriptive statistics were calculated as medians and IQRs for continuous variables and as counts and percentages for categorical variables. Distributions of continuous variables were illustrated with kernel density plots. The correlation between EHR-derived workload and billing-derived workload was assessed using Pearson correlation coefficient, both for individual encounters and after averaging over each anesthesia *CPT* code in the cohort. In the *CPT*-averaged analysis, only *CPT* codes with at least 50 individual encounters were used to generate reliable average measures of EHR-derived and billing-derived workload.

To evaluate the differential estimated effect of patient complexity, procedure complexity, and anesthesia duration on the EHR-derived measure of anesthesiology workload compared with the billing-derived measure of workload, we restructured the data to include 2 observations per encounter (1 observation for each measure of workload) and then constructed a single multivariable mixed-effect linear regression model. The model included the ASA modifier, procedural base units, and anesthesia time units as independent variables and the encounter-level total count of EHR actions and total billed units as the 2 outcome variables. To directly compare the 2 workload measures in the same model, these variables were standardized to *z* scores with a mean of 0 and an SD of 1. Interaction terms between a dummy variable (representing the type of workload outcome) and the independent variables were used to determine whether the associations varied differentially by workload type. The resulting β coefficients were interpreted as the expected effect of a 1-unit change in each independent variable on the standardized workload outcome. For example, a β coefficient of 0.1 means that a 1-unit change in the specified independent variable results in a 0.1-SD change in the specified workload outcome.

We determined, for each independent variable, whether the β coefficient for the EHR-derived workload outcome differed significantly from that of the billing-derived workload outcome; we interpreted this as a differential contribution of the independent variable toward each workload outcome. Therefore, custom hypothesis tests were specified to evaluate statistical significance of the difference in these 2 β coefficients.

*P* values were 2-tailed, and a *P* value less than .05 was considered to be statistically significant. Statistical analyses were performed in SAS version 9.4 (SAS Institute). Additional details on the data restructuring and statistical model specification can be found in the eMethods in Supplement 1.

## Results

### Characteristics of the Study Cohort

A total of 405 anesthesiology clinicians (146 anesthesiologists, 161 nurse anesthetists, 41 resident physicians, and 57 other health care professionals) working across 8 hospitals (2 academic, 4 community, and 2 outpatient surgical centers) were included in the study. During the study period, these clinicians provided anesthetic services for 31 688 surgical encounters and performed a total of 8 288 132 EHR-based actions encompassing 39 131 hours of EHR use. General characteristics of the anesthetic encounters are shown in the Table.

**Table.** Characteristics of the Included Anesthesiology Clinicians and Associated Anesthesia Encounters During the Study Period

Characteristic	Encounters, No. (%)
Involved clinician	
Anesthesiologist	31 661 (100)
Nurse anesthetist	23 088 (73)
Resident	3850 (12)
Other	3418 (11)
Encounter location	
Academic hospital	22 267 (70)
Community hospital	6174 (20)
Surgical center	3246 (10)
Missing	1 (<1)
Disposition	
Outpatient	20 027 (63)
Inpatient	9354 (29)
Missing	2317 (7)
Encounter age category	
Adult	25 006 (79)
Pediatric	6682 (21)
ASA physical status score	
1	3816 (12)
2	14 769 (47)
3	11 564 (36)
4	1501 (5)
5	35 (0.1)
Procedural base units	
3	5055 (17)
4	4945 (16)
5	6897 (22)
6	3820 (12)
7	4907 (15)
≥8	4734 (15)
Anesthesia duration, h	
<1	8145 (26)
1-<2	10 295 (32)
2-<3	6293 (20)
3-<4	3192 (10)
≥4	3763 (12)
Surgical service	
Orthopedics	7581 (24)
General surgery	3733 (12)
Ophthalmology	3220 (10)
Head and neck	3163 (10)
Urology	2418 (8)
Obstetrics/gynecology	2265 (7)
Cardiovascular	2015 (6)
Plastic surgery	1374 (4)
Neurosurgery	1264 (4)
Other	2346 (7)
Missing	2317 (7)

Abbreviation: ASA, American Society of Anesthesiology.

### EHR Use Stratified by Phase of Care

**Figure 1.**
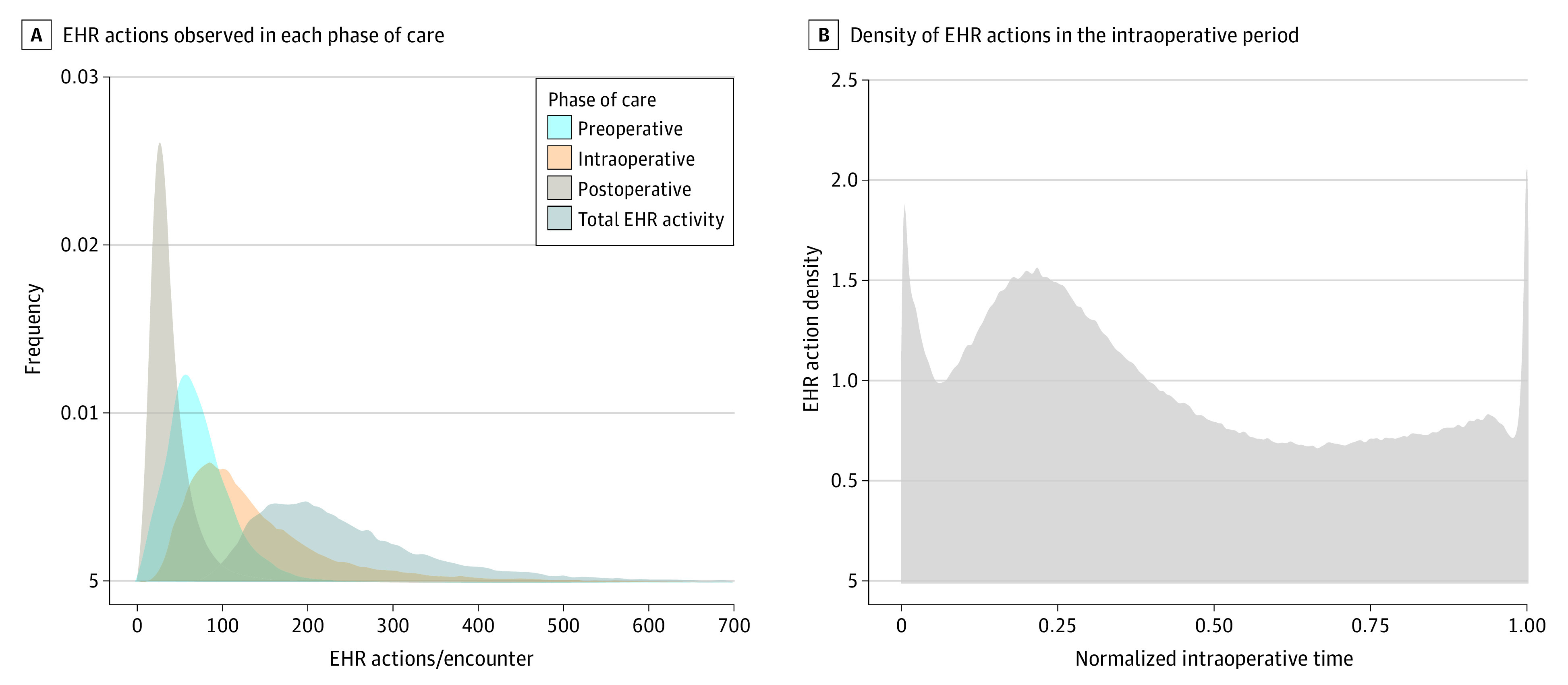
Distribution of Electronic Health Record (EHR) Activity by Phase of Care Across All Anesthetic Encounters A, Kernel density histogram illustrating the number of EHR actions observed in each phase of care for all anesthetic encounters included in the study. The y-axis is shown in arbitrary units, indicating the density of observations in each bin. B, Kernel density histogram illustrating the density of EHR actions in the intraoperative period relative to the start and end of the anesthetic. Time on the x-axis is normalized such that 0 is the start of the anesthetic and 1 corresponds to the end of the anesthetic.

For each encounter, a median (IQR) of 224 (168-308) EHR-based actions were performed across all anesthesiology clinicians caring for the patient during the perioperative period (Figure 1A). A median (IQR) of 117 (79-180) actions per encounter were observed during the intraoperative period (Figure 1A). Intraoperative actions typically involved the documentation of medication administrations and intraoperative events. The highest density of EHR-based actions occurred at the start and end of the encounter and following induction of anesthesia (Figure 1B).

Nearly as much EHR use occurred in the preoperative period (Figure 1A) as the intraoperative period. For each encounter, clinicians spent a median (IQR) of 11 (6-19) minutes using the EHR and performing a median (IQR) of 66 (45-92) EHR-based actions in the preoperative period. Preoperative actions typically involved review of patient data. Most preoperative EHR use occurred in the minutes to hours leading up to the start of each encounter (eFigure 3 in Supplement 1); however, 15 505 of 31 688 encounters (48.9%) also had at least 1 clinician viewing the EHR at least 12 hours prior to anesthesia start, illustrating that preoperative preparation for anesthesia often begins the day before surgery.

Compared with the other phases of care, the postoperative period involved less EHR activity (median [IQR] of 31 [22-46] actions per encounter) (Figure 1A). Most postoperative activity occurred in the minutes following anesthesia stop as clinicians completed their postanesthetic EHR logging and provided additional care in the recovery unit (eFigure 4 in Supplement 1). A total of 6449 of 31 688 encounters (20.4%) had activity occurring more than 24 hours after anesthesia stop. This delayed activity represented both follow-up of the patient’s clinical status and delayed completion of required documentation.

### Comparison of EHR-Derived Workload With Patient and Procedural Complexity

**Figure 2.**
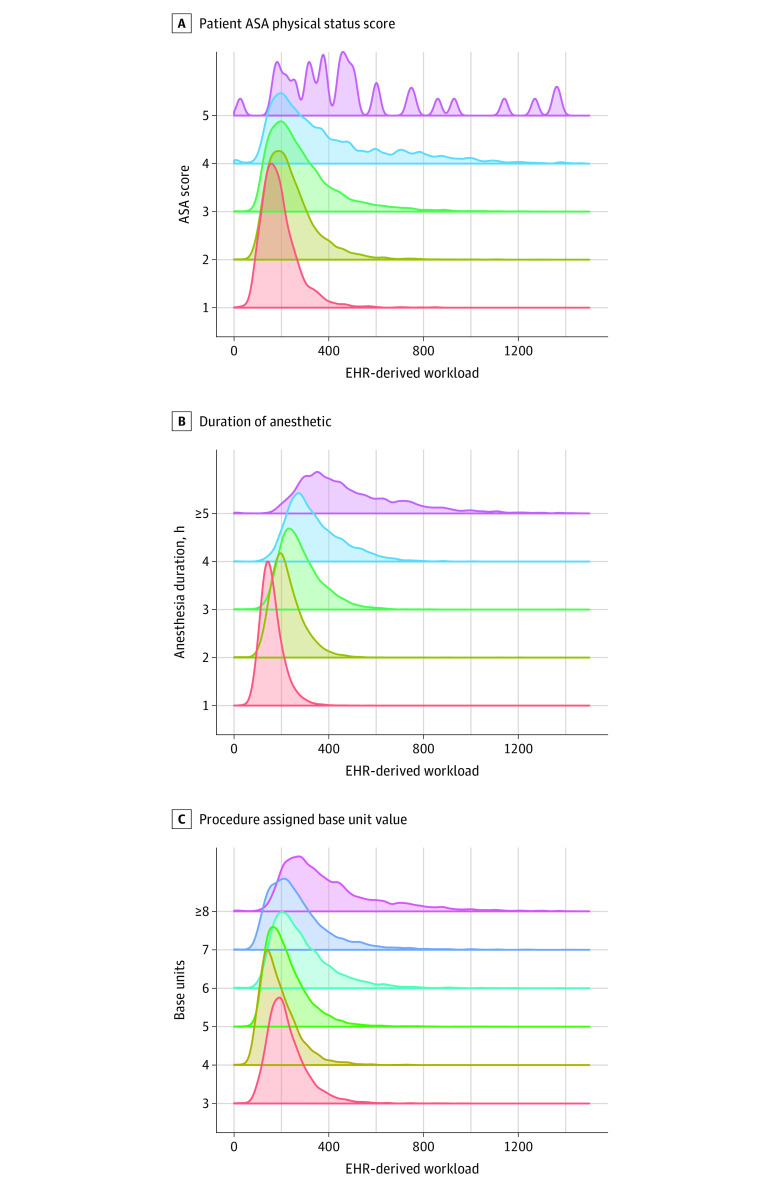
Association Between Electronic Health Record (EHR)—Derived Workload and Patient Complexity, Procedure Complexity, and Anesthesia Duration Kernel density histograms illustrating the distribution of the total number of EHR actions observed for the encounter, (A) stratified by the patient’s American Society of Anesthesiology (ASA) physical status score as a measure of patient complexity, (B) stratified by the duration of the anesthetic in hours, and (C) stratified by the procedure’s assigned base unit value according to the ASA Relative Value Guide^18^ as a measure of procedure complexity.

We examined the association between EHR-derived workload and other known measures of anesthesiology workload, such as patient complexity, procedure complexity, and anesthesia duration. On average, EHR-derived workload increased as patient complexity increased (Figure 2A). For example, the median (IQR) number of EHR actions was 179 (140-229) vs 218 (167-288) for patients with an ASA physical status score of 1 vs 2, respectively. The median EHR actions also increased as anesthesia duration increased (Figure 2B). For example, a median (IQR) of 151 (127-182) actions were observed during encounters lasting less than 1 hour compared with 433 (333-607) actions for encounters lasting more than 4 hours.

However, the association between EHR-derived workload and procedural base units was less clear (Figure 2C). Although total EHR activity did generally increase as the number of base units increased, median activity did not always increase between each base unit increment. For example, median (IQR) EHR activity was 206 (166-228) actions for 3 base unit encounters compared with 171 (133-228) actions for 4 base unit encounters.

### Multivariable Model for Anesthesiology Workload

**Figure 3.**
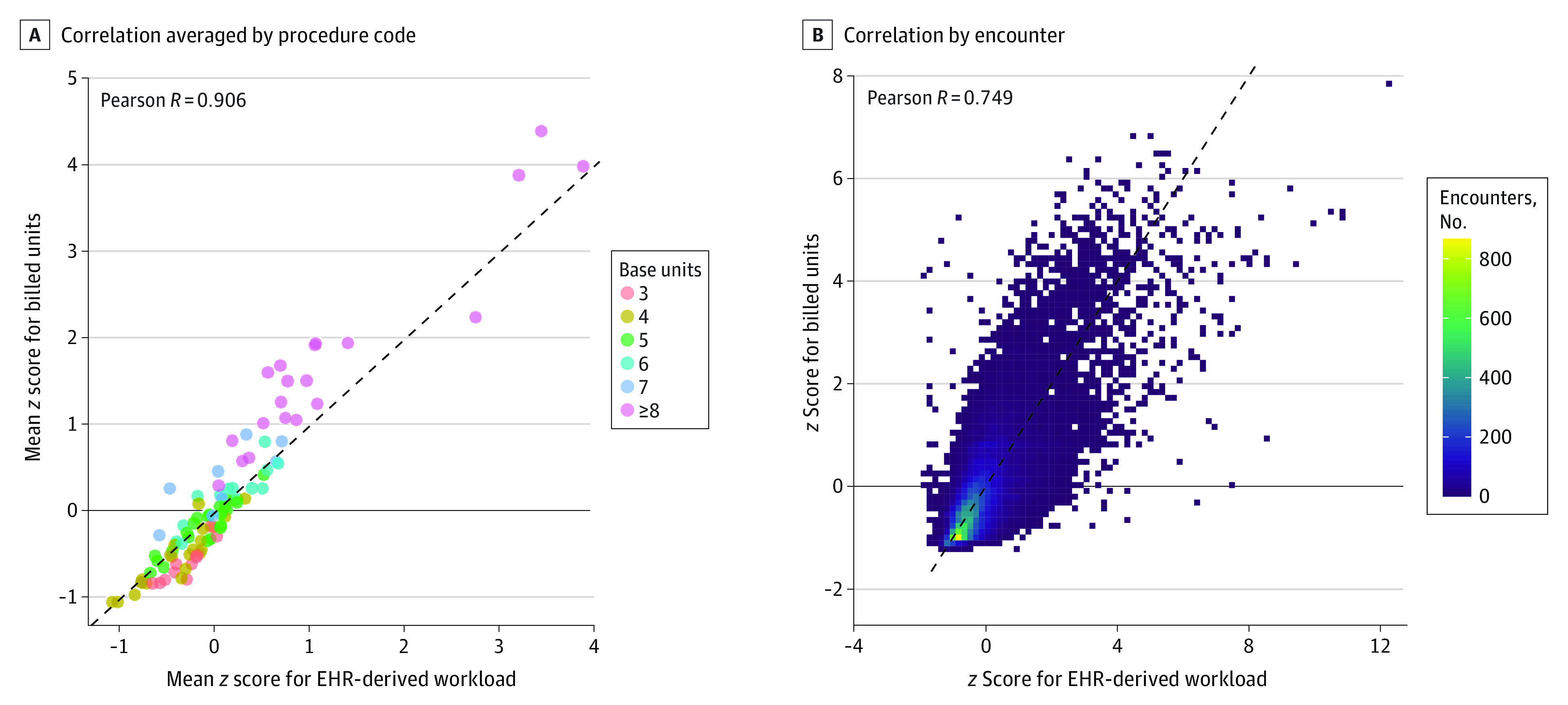
Correlation Between Electronic Health Record (EHR)—Derived Workload and Billing-Derived Workload A, For each anesthesia *Current Procedural Terminology* code, the mean number of EHR actions observed and mean number of units billed was plotted, with a single dot for each procedure code, colored by that procedure code’s base unit value. Only anesthesia *Current Procedural Terminology* codes with at least 50 encounters are shown; this included 29 747 of 31 688 encounters (94%). See eTable 2 in Supplement 1 for a list of all anesthesia *Current Procedural Terminology* codes included. B, For each encounter, the number of EHR actions observed and number of units billed were plotted. Due to the large number of encounters, a 2-dimensional histogram is shown, with color indicating the number of encounters in each bin.

We investigated the association between total billed units (the billing-derived measure of anesthesiology workload) and total EHR activity (our EHR-derived measure). Averaged across each anesthesia *CPT* code, mean billed units was highly correlated with mean EHR actions (Pearson *R* = 0.906; Figure 3A). However, there was considerable variation at the individual-encounter level (Pearson *R* = 0.749; Figure 3B).

**Figure 4.**
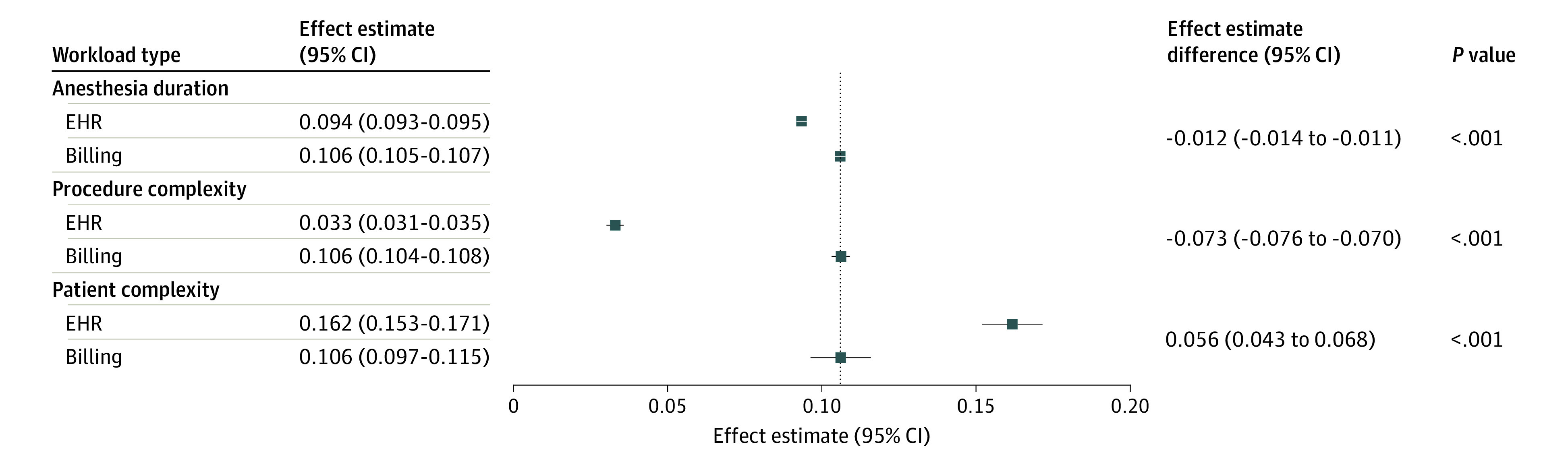
Relative Contribution of Anesthesia Duration, Patient Complexity, and Procedure Complexity to Electronic Health Record (EHR)—Derived and Billing-Derived Workload A linear mixed-effects model was used to measure the contribution of patient complexity, procedure complexity, and anesthesia duration to the 2 outcome variables: standardized EHR-derived workload and standardized billing-derived workload. The effect estimates refer to the contribution of a 1-unit change in each independent variable toward each standardized outcome variable. Because billed units are defined as the sum of time units, the American Society of Anesthesiology modifier, and procedure base units, the coefficients fit for these independent variables towards the billed unit outcome were expected to be equal. The primary goal of this analysis was to examine the relative value of the estimate for the EHR-derived outcome compared with the billing-derived outcome, which was interpreted as relative difference in contribution of each independent variable toward each outcome.

To explore how EHR-derived and billing-derived workload differed at the individual-encounter level, we constructed a single multivariable model for both measures of workload as a function of ASA modifier, procedural base units, and time units (Figure 4). For each independent variable, the difference in coefficient between the billing-derived outcome and the EHR-derived outcome reflected the relative difference in contribution of that variable to each outcome; these differences were all statistically significant. For example, the contribution of anesthesia duration toward the EHR-derived measure of workload (β = 0.094; 95% CI, 0.093-0.095) was slightly less than its contribution to the billing-derived measure of workload (β = 0.106; 95% CI, 0.105-0.107) (*P* for difference < .001). In contrast, the contribution of patient complexity toward EHR-derived workload (β = 0.162; 95% CI, 0.153-0.171) was more than 50% greater than its contribution toward billing-derived workload (β = 0.106; 95% CI, 0.097-0.116) (*P* for difference < .001). In addition, the contribution of procedural complexity toward EHR-derived workload (β = 0.033; 95% CI, 0.031-0.035) was approximately one-third its contribution toward billing-derived workload (β = 0.106; 95% CI, 0.104-0.108) (*P* for difference < .001). It should be noted that the coefficients for each of the independent variables toward billing-derived workload were all equal by design, as patient complexity, procedural complexity, and time contribute 1:1:1 to the definition of billed units. Subgroup analysis stratified by academic vs community settings showed similar results (eTable 1 in Supplement 1).

## Discussion

In this study, we developed a scalable approach to measure clinical workload in anesthesiology using EHR audit log data. The following findings support the viability of our approach: (1) our EHR-derived measure of intraoperative workload was consistent with previous time-motion studies of intraoperative anesthesia workload in that workload was highest during induction and emergence from anesthesia (Figure 1B)^20,21^; (2) EHR-derived workload scaled with known contributors to workload, such as patient complexity, procedural complexity, and time (Figure 2); and (3) at the procedural level, average EHR-derived workload was highly correlated with average billed RVUs (Figure 3A).

However, at the individual-encounter level, there was substantial variation in EHR-derived workload compared with billing-derived workload (Figure 3B). In multivariable analysis, patient complexity contributed substantially more to EHR-derived workload than billing-derived workload; conversely, procedural complexity contributed much less to EHR-derived workload than billing-derived workload (Figure 4). If we consider EHR-derived workload as a reasonable proxy for actual clinical workload, these findings suggest that anesthesia reimbursement may be poorly calibrated with clinical workload. In other words, anesthesiologists are relatively undercompensated for patients with more severe illness having minor procedures and overcompensated for healthy patients having major procedures. This is especially true for patients with public insurance, who make up approximately one-half of total anesthetic volume,^22^ as CMS does not reimburse for the ASA physical status modifier. In addition, this discrepancy likely disproportionately affects academic medical centers and safety net hospitals that care for more complex patients.^23^ Our findings are in line with recent findings that the CMS RVU schedule for surgical reimbursement also likely undercompensates for patient complexity.^10^

Our results highlight several potential flaws with the current reimbursement system for anesthetic services in the US. Unlike reimbursement for surgical services, anesthesia reimbursement does not explicitly account for preoperative or postoperative care. As has been argued for other medical specialties,^24,25,26^ our results suggest that the current system does not appropriately reward the cognitive work of anesthesia, which largely occurs during the preoperative period.^27,28^ Instead, such work is currently bundled into the procedural base rate. Our results also suggest that these base units may be miscalibrated, especially at the low end; for example, the EHR-derived workload for 3 base unit procedures was on average higher than for 4 base unit procedures (Figure 2B).

Our approach for measuring clinical workload from EHR log data has potential to be broadly applicable for other specialties. The CMS RVU system has been widely criticized for its reliance on subjective survey data to measure the time and intensity of clinical work.^7,8,11^ Our method offers opportunities to quantitatively measure both time and intensity across large clinician populations, which could be useful to influence future revisions to reimbursement policy.^6,29^

### Limitations

This study has several limitations. We rely on the assumption that EHR activity is proportional to actual clinical work. Anesthesiology is relatively unique in that direct patient care does not require the EHR; however, clinical care is expected to be documented in the EHR, and previous time-motion studies have shown that anesthesiologists spend approximately one-quarter of their time interacting with the EHR.^30^ The administration and titration of medications, fluids, and blood products are largely represented in the audit log data. However, not all anesthetic work is captured; for example, verbal communication with the surgical team, physical movements such as patient transport or making ventilator changes, and the overall maintenance of vigilance are not captured. In addition, the work of performing procedures (such as arterial lines or transesophageal echocardiography) is likely underrepresented in the audit log data (where they appear as a procedure note requiring relatively few EHR actions to generate); however, such procedures are typically billed separately and thus are reasonable to not include in our comparison with standard anesthesia reimbursement.

This study was conducted across a single health system with a shared EHR, and the results may not generalize to other health systems or specialties. Although a diverse range of hospitals are included (including academic and community, adult and pediatric, and urban and rural), the primary mode of anesthesia provision is under medical direction (eg, with 1 attending anesthesiologist and 1 secondary health care professional, such as a nurse anesthetist), with relatively few encounters under nonmedical direction or personally performed. Different EHR-derived workload measurements may occur with different supervision ratios. Due to our supervision ratio, we were unable to adjust for health care professional—level clustering in EHR use in our statistical approach, as multiple professionals participated in each anesthetic encounter.

## Conclusions

In this cross-sectional study of 8 hospitals in 2 states, we found that reimbursement for anesthesiology services overcompensated for procedural complexity and undercompensated for patient complexity. Our results suggest that the current system for anesthesiology reimbursement might be poorly calibrated with clinical workload. The method for measuring clinical workload developed in this study could be used to improve reimbursement valuations.

## Notes

Supplement 1.
eMethods.
**eFigure 1.** Example Audit Log, Illustrating Some Typical Actions Recorded During an Encounter **eFigure 2.** CONSORT Diagram for Encounter Inclusion and Exclusion in the Study **eFigure 3.** Distribution of the Time That EHR Actions in the Preoperative Period Were Performed Relative to the Start of the Intraoperative Period (Anesthesia Start) **eFigure 4.** Distribution of the Time that EHR Actions in the Postoperative Period Were Performed Relative to the End of the Intraoperative Period (Anesthesia Stop) **eTable 1.** Association Between Patient Complexity, Procedure Complexity, and Anesthesia Duration and EHR-Derived vs Billing-Derived Workload, Stratified by Setting (Academic Hospital vs Community Hospital and Surgical Center) **eTable 2.** List of All Anesthesia *CPT* Codes Included in Figure 3A

Supplement 2.
Data Sharing Statement
