# Supplementary material for: Anesthesia Clinical Workload Estimated From Electronic Health Record Documentation vs Billed Relative Value Units
Source: JAMA Netw Open. 2023 Aug 11;6(8):e2328514. doi: 10.1001/jamanetworkopen.2023.28514 (PMC10422189; doi:10.1001/jamanetworkopen.2023.28514)
Supplement: Supplement 1. — eMethods. eFigure 1. Example Audit Log, Illustrating Some Typical Actions Recorded During an Encounter eFigure 2. CONSORT Diagram for Encounter Inclusion and Exclusion in the Study eFigure 3. Distribution of the Time That EHR Actions in the Preoperative Period Were Performed Relative to the Start of the Intraoperative Period (Anesthesia Start) eFigure 4. Distribution of the Time that EHR Actions in the Postoperative Period Were Performed Relative to the End of the Intraoperative Period (Anesthesia Stop) eTable 1. Association Between Patient Complexity, Procedure Complexity, and Anesthesia Duration and EHR-Derived vs Billing-Derived Workload, Stratified by Setting (Academic Hospital vs Community Hospital and Surgical Center) eTable 2. List of All Anesthesia CPT Codes Included in Figure 3A [file jamanetwopen-e2328514-s001.pdf]

Supplemental Online Content

Lou SS, Baratta LR, Lew D, Harford D, Avidan MS, Kannampallil T. Anesthesia clinical workload estimated from electronic health record documentation vs billed relative value units. *JAMA Netw Open*. 2023;6(8):e2328514. doi:10.1001/jamanetworkopen.2023.28514

eMethods.
eFigure 1. Example Audit Log, Illustrating Some Typical Actions Recorded During an Encounter
eFigure 2. CONSORT Diagram for Encounter Inclusion and Exclusion in the Study
eFigure 3. Distribution of the Time That EHR Actions in the Preoperative Period Were Performed Relative to the Start of the Intraoperative Period (Anesthesia Start)
eFigure 4. Distribution of the Time that EHR Actions in the Postoperative Period Were Performed Relative to the End of the Intraoperative Period (Anesthesia Stop)
eTable 1. Association Between Patient Complexity, Procedure Complexity, and Anesthesia Duration and EHR-Derived vs Billing-Derived Workload, Stratified by Setting (Academic Hospital vs Community Hospital and Surgical Center)
eTable 2. List of All Anesthesia *CPT* Codes Included in Figure 3A

This supplementary material has been provided by the authors to give readers additional information about their work.

© 2023 Lou SS et al. *JAMA Network Open*.

eMethods.

Data Pre-Processing

To directly compare the relationships between case duration, case complexity, and patient complexity on the two measures of workload in a single statistical model, both workload measures were standardized to a common distribution. Specifically, the variables for billed units and total EHR activity were standardized to z-scores for the total population of procedures included in the analysis. As expected with z-scores, the new distributions for each workload measure had a mean of zero and standard deviation of one.

In the resulting transformed dataset, each encounter had two outcome measures, a standardized measure of EHR-derived workload and a standardized measure of billing-derived workload. To facilitate comparison of these two outcomes in a single statistical model, a long-form dataset was created such that each encounter was represented twice, once with the billing-derived measure of workload and once with the EHR-derived measure of workload.

Both observations had the same values for case duration, procedure complexity, and patient complexity, as these were encounter-level variables. The two observations differed in their measurement of workload, with the first observation having a workload z-score derived from procedure quantity whereas the second observation had a workload z-score derived from total intraoperative clicks. A dummy variable indicating the source of the workload measure (EHR-derived versus billing-derived) was also created and added to each observation in the dataset.

Below is an example of what the data looked like following the pre-processing:

Encounter ID	Workload (z-score)	Workload Source	Time Units	Procedure Complexity	Patient Complexity
121	-0.23	Billed Units	14	7	0
121	-0.52	EHR derived	14	7	0
122	0.78	Billed Units	22	9	2
122	0.42	EHR derived	22	9	2
123	0.11	Billed Units	6	4	1
123	-0.05	EHR derived	6	4	1

Statistical Analysis

A mixed-effects linear regression model was used to examine the relationship between the independent variables of interest and workload, with the equation as follows:

$$\begin{aligned} Workload_i = \; & \beta_{0i} + \beta_1(Case\ Duration) + \beta_2(Patient\ Complexity) + \beta_3(Procedure\ Complexity) \\ & + \beta_4(Workload\ Source) + \beta_{1,4}(Case\ Duration * Workload\ Source) \\ & + \beta_{2,4}(Patient\ Complexity * Workload\ Source) \\ & + \beta_{3,4}(Procedure\ Complexity * Workload\ Source) + e_i \end{aligned}$$

Random intercepts (β_{0i}) were used to account for the two repeated observations, *i*, for each encounter. The dependent variable $Workload_i$ refers to the workload measurement based on either billing or EHR activity, while the $Workload\ Source$ term refers to the dummy variable indicating these two sources.

© 2023 Lou SS et al. *JAMA Network Open*.

Interaction terms between this dummy variable representing the workload source and the independent variables of case duration, patient complexity, and procedure complexity were included in the model, as shown in the equation above. Restructuring the data to include two observations per encounter and incorporating these interaction terms into the model allowed us to run one model to examine the relationships between the independent variables of interest and each workload source simultaneously, as opposed to two separate models using each workload source as separate dependent variables. The advantage to using one model, as opposed to two separate ones, was that it allowed us to directly compare the effect estimates to one another. Specifically, we were able to use custom hypothesis test statements to estimate the relationship between the independent variables and each workload source, as well as the difference between the effect estimates for each workload source. This allowed us to directly test the hypotheses of whether the effect estimates for each workload source were significantly different from one another, which would not have been possible had we used two separate regression equations. An example of how the custom hypothesis tests were specified is shown below:

- Effect of case duration on billed units was calculated as $\beta_1 + \beta_{1,4}(Workload\ source = billed\ units)$
- Effect of case duration on EHR activity was calculated as $\beta_1 + \beta_{1,4}(Workload\ source = EHR\ derived)$
- Difference in effect of case duration on billed units versus EHR activity was calculated as $\beta_{1,4}(Workload\ source = billed\ units) - \beta_{1,4}(Workload\ source = EHR\ derived)$

Statistical analyses were performed using the MIXED procedure in SAS 9.4 and SAS Enterprise Guide 8.3.

© 2023 Lou SS et al. *JAMA Network Open*.

eFigure 1. Example Audit Log, Illustrating Some Typical Actions Recorded During an Encounter

ACCESS_TIME indicates the timestamp when the action was performed in the EHR. METRIC_NAME and REPORT_NAME provide information on the type of action that was performed. PAT_ID indicates the patient identifier on which the action occurred. USER_ID indicates the user performing the action.

ACCESS_TIME	METRIC_NAME	REPORT_NAME	PAT_ID	USER_ID
10/21/19 6:36	App Report Viewed			Z
10/21/19 6:36	Storyboard viewed	AN Anesthesia Storyboard	A	Z
10/21/19 6:37	Visit Navigator template loaded		A	Z
10/21/19 6:37	Report with patient data viewed	AN PROCEDURE INFO REPORT W/CPT	A	Z
10/21/19 6:37	Report with patient data viewed	AN Relevant Encounters	A	Z
10/21/19 6:39	Intraprocedure viewed		A	Z
10/21/19 6:39	Intraprocedure sidebar report viewed	BW AN Intraprocedure Sidebar Report	A	Z
10/21/19 7:00	Intraprocedure Staff viewed		A	Z
10/21/19 7:00	Intraprocedure Staff edited		A	Z
10/21/19 7:00	Intraprocedure Attestations viewed		A	Z
10/21/19 7:00	Report with patient data viewed	BW AN OTHER PROVIDER ATTESTATION REPORT	A	Z
10/21/19 7:00	Attestation recorded in Intraprocedure		A	Z
10/21/19 7:31	Patient device added		A	Z
10/21/19 7:31	Intraprocedure Event edited		A	Z
10/21/19 7:43	Intraprocedure Meds viewed		A	Z
10/21/19 7:43	Intraprocedure Meds edited		A	Z
10/21/19 7:48	Intraprocedure Flowsheets viewed		A	Z
10/21/19 7:48	Intraprocedure Flowsheets edited		A	Z
10/21/19 7:50	Clinical Note Signed	Anesthesia Intubation Note	A	Z
10/21/19 7:50	LDA created via procedure documentation		A	Z

© 2023 Lou SS et al. *JAMA Network Open*.

eFigure 2. CONSORT Diagram for Encounter Inclusion and Exclusion in the Study

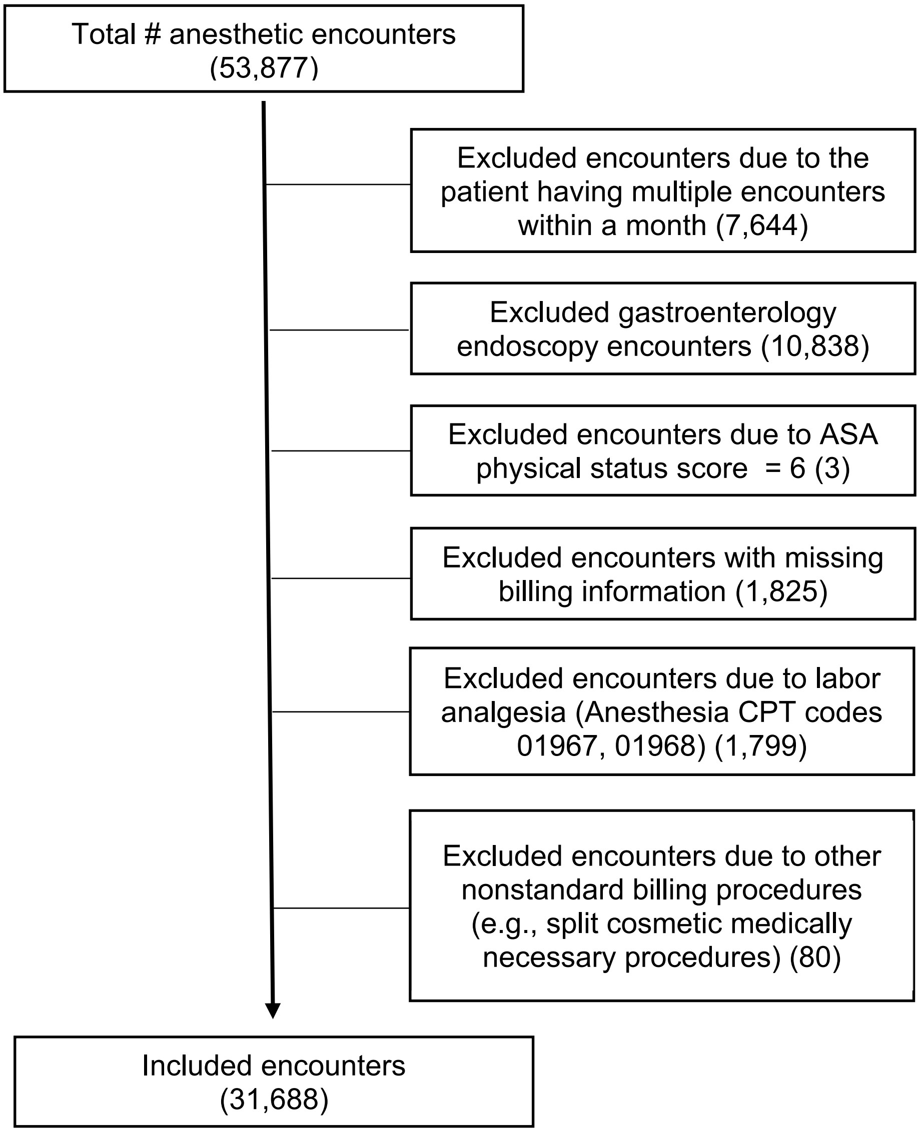

© 2023 Lou SS et al. *JAMA Network Open*.

eFigure 3. Distribution of the Time That EHR Actions in the Preoperative Period Were Performed Relative to the Start of the Intraoperative Period (Anesthesia Start)

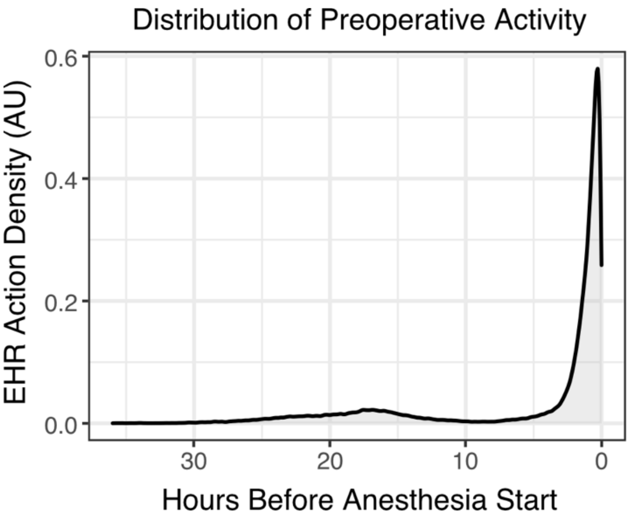

© 2023 Lou SS et al. *JAMA Network Open*.

eFigure 4. Distribution of the Time that EHR Actions in the Postoperative Period Were Performed Relative to the End of the Intraoperative Period (Anesthesia Stop)

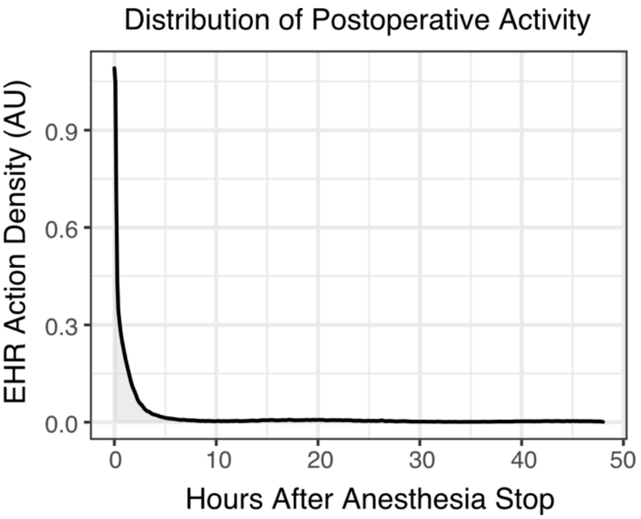

© 2023 Lou SS et al. *JAMA Network Open*.

eTable 1. Association Between Patient Complexity, Procedure Complexity, and Anesthesia Duration and EHR-Derived vs Billing-Derived Workload, Stratified by Setting (Academic Hospital vs Community Hospital and Surgical Center)

Z-scores for the two outcome variables (total EHR actions and billed units) were computed separately for academic vs community settings to assess whether the contribution of the independent variables (anesthesia duration, patient complexity, procedure complexity) toward each outcome variable differed by setting. A single linear mixed-effect model was used, similar to that described in the main manuscript, except a three-way interaction effect was included between the independent variable of interest, the type of workload measure (EHR-derived vs billing-derived), and the setting (academic vs. community). Using this three-way interaction, custom hypothesis test estimates were specified to determine the relationship between the independent variables and the outcomes, stratified by setting. All effect estimates were statistically significant ($p < 0.001$). The p-values shown refer to the comparison between the effect estimate for academic setting and the community setting for each independent variable.

**Independent Variable**	**Effect on Billed Units (95% CI)**	**Effect on Total EHR Actions – Academic (95% CI)**	**Effect on Total EHR Actions – Community (95% CI)**	**P-value** (acad. vs. community)
**Time units** (Anesthesia duration)	0.106 (0.105, 0.107)	0.084 (0.083, 0.085)	0.165 (0.162, 0.168)	< 0.001
**ASA modifier** (Patient complexity)	0.106 (0.097, 0.116)	0.163 (0.153, 0.174)	0.144 (0.123, 0.165)	0.11
**Procedure base units** (Procedure complexity)	0.106 (0.104, 0.108)	0.033 (0.030, 0.035)	0.022 (0.016, 0.028)	< 0.001

© 2023 Lou SS et al. *JAMA Network Open*.

eTable 2. List of All Anesthesia *CPT* Codes Included in Figure 3A

Anesthesia CPT Code	Descriptor	Count
01922	Anesthesia for non-invasive imaging or radiation therapy	1522
00840	Anesthesia for intraperitoneal procedures in lower abdomen including laparoscopy; not otherwise specified	1415
00140	Anesthesia for procedures on eye; not otherwise specified	1275
00400	Anesthesia for procedures on the integumentary system on the extremities, anterior trunk and perineum; not otherwise specified	1236
00790	Anesthesia for intraperitoneal procedures in upper abdomen including laparoscopy; not otherwise specified	1234
00142	Anesthesia for procedures on eye; lens surgery	1086
01810	Anesthesia for all procedures on nerves, muscles, tendons, fascia, and bursae of forearm, wrist, and hand	893
00170	Anesthesia for intraoral procedures, including biopsy; not otherwise specified	842
00300	Anesthesia for all procedures on the integumentary system, muscles and nerves of head, neck, and posterior trunk, not otherwise specified	839
01402	Anesthesia for open or surgical arthroscopic procedures on knee joint; total knee arthroplasty	802
00670	Anesthesia for extensive spine and spinal cord procedures (eg, spinal instrumentation or vascular procedures)	789
01400	Anesthesia for open or surgical arthroscopic procedures on knee joint; not otherwise specified	781
00320	Anesthesia for all procedures on esophagus, thyroid, larynx, trachea and lymphatic system of neck; not otherwise specified, age 1 year or older	758
01480	Anesthesia for open procedures on bones of lower leg, ankle, and foot; not otherwise specified	748
01961	Anesthesia for cesarean delivery only	620
01214	Anesthesia for open procedures involving hip joint; total hip arthroplasty	573
01830	Anesthesia for open or surgical arthroscopic/endoscopic procedures on distal radius, distal ulna, wrist, or hand joints; not otherwise specified	568
00902	Anesthesia for; anorectal procedure	562
00126	Anesthesia for procedures on external, middle, and inner ear including biopsy; tympanotomy	541
00910	Anesthesia for transurethral procedures (including urethrocystoscopy); not otherwise specified	493

© 2023 Lou SS et al. *JAMA Network Open*.

00402	Anesthesia for procedures on the integumentary system on the extremities, anterior trunk and perineum; reconstructive procedures on breast (eg, reduction or augmentation mammoplasty, muscle flaps)	474
01630	Anesthesia for open or surgical arthroscopic procedures on humeral head and neck, sternoclavicular joint, acromioclavicular joint, and shoulder joint; not otherwise specified	453
00940	Anesthesia for vaginal procedures (including biopsy of labia, vagina, cervix or endometrium); not otherwise specified	351
00160	Anesthesia for procedures on nose and accessory sinuses; not otherwise specified	349
01638	Anesthesia for open or surgical arthroscopic procedures on humeral head and neck, sternoclavicular joint, acromioclavicular joint, and shoulder joint; total shoulder replacement	336
00145	Anesthesia for procedures on eye; vitreoretinal surgery	335
00210	Anesthesia for intracranial procedures; not otherwise specified	319
00103	Anesthesia for reconstructive procedures of eyelid (eg, blepharoplasty, ptosis surgery)	290
00952	Anesthesia for vaginal procedures (including biopsy of labia, vagina, cervix or endometrium); hysteroscopy and/or hysterosalpingography	284
00862	Anesthesia for extraperitoneal procedures in lower abdomen, including urinary tract; renal procedures, including upper one-third of ureter, or donor nephrectomy	281
01926	Anesthesia for therapeutic interventional radiological procedures involving the arterial system; intracranial, intracardiac, or aortic	261
01610	Anesthesia for all procedures on nerves, muscles, tendons, fascia, and bursae of shoulder and axilla	240
01470	Anesthesia for procedures on nerves, muscles, tendons, and fascia of lower leg, ankle, and foot; not otherwise specified	238
00120	Anesthesia for procedures on external, middle, and inner ear including biopsy; not otherwise specified	235
00520	Anesthesia for closed chest procedures; (including bronchoscopy) not otherwise specified	229
00797	Anesthesia for intraperitoneal procedures in upper abdomen including laparoscopy; gastric restrictive procedure for morbid obesity	226
00830	Anesthesia for hernia repairs in lower abdomen; not otherwise specified	226
00918	Anesthesia for transurethral procedures (including urethrocystoscopy); with fragmentation, manipulation and/or removal of ureteral calculus	224
00920	Anesthesia for procedures on male genitalia (including open urethral procedures); not otherwise specified	216

© 2023 Lou SS et al. *JAMA Network Open*.

01230	Anesthesia for open procedures involving upper two-thirds of femur; not otherwise specified	210
00630	Anesthesia for procedures in lumbar region; not otherwise specified	207
01844	Anesthesia for vascular shunt, or shunt revision, any type (eg, dialysis)	199
00865	Anesthesia for extraperitoneal procedures in lower abdomen, including urinary tract; radical prostatectomy (suprapubic, retropubic)	198
00860	Anesthesia for extraperitoneal procedures in lower abdomen, including urinary tract; not otherwise specified	194
00811	Anesthesia for lower intestinal endoscopic procedures, endoscope introduced distal to duodenum; not otherwise specified	192
00731	Anesthesia for upper gastrointestinal endoscopic procedures, endoscope introduced proximal to duodenum; not otherwise specified	191
00537	Anesthesia for cardiac electrophysiologic procedures including radiofrequency ablation	190
00410	Anesthesia for procedures on the integumentary system on the extremities, anterior trunk and perineum; electrical conversion of arrhythmias	180
00812	Anesthesia for lower intestinal endoscopic procedures, endoscope introduced distal to duodenum; screening colonoscopy	166
00190	Anesthesia for procedures on facial bones or skull; not otherwise specified	161
00534	Anesthesia for transvenous insertion or replacement of pacing cardioverter-defibrillator	161
00541	Anesthesia for thoracotomy procedures involving lungs, pleura, diaphragm, and mediastinum (including surgical thoracoscopy); utilizing 1 lung ventilation	156
00560	Anesthesia for procedures on heart, pericardial sac, and great vessels of chest; without pump oxygenator	151
01740	Anesthesia for open or surgical arthroscopic procedures of the elbow; not otherwise specified	145
00912	Anesthesia for transurethral procedures (including urethrocystoscopy); transurethral resection of bladder tumor(s)	143
01210	Anesthesia for open procedures involving hip joint; not otherwise specified	140
00635	Anesthesia for procedures in lumbar region; diagnostic or therapeutic lumbar puncture	139
01920	Anesthesia for cardiac catheterization including coronary angiography and ventriculography (not to include Swan-Ganz catheter)	137
00868	Anesthesia for extraperitoneal procedures in lower abdomen, including urinary tract; renal transplant (recipient)	130
01710	Anesthesia for procedures on nerves, muscles, tendons, fascia, and bursae of upper arm and elbow; not otherwise specified	130

© 2023 Lou SS et al. *JAMA Network Open*.

00470	Anesthesia for partial rib resection; not otherwise specified	123
01360	Anesthesia for all open procedures on lower one-third of femur	121
00914	Anesthesia for transurethral procedures (including urethrocystoscopy); transurethral resection of prostate	119
00532	Anesthesia for access to central venous circulation	116
01392	Anesthesia for all open procedures on upper ends of tibia, fibula, and/or patella	116
01250	Anesthesia for all procedures on nerves, muscles, tendons, fascia, and bursae of upper leg	112
00104	Anesthesia for electroconvulsive therapy	110
01202	Anesthesia for arthroscopic procedures of hip joint	110
00530	Anesthesia for permanent transvenous pacemaker insertion	108
00562	Anesthesia for procedures on heart, pericardial sac, and great vessels of chest; with pump oxygenator, age 1 year or older, for all noncoronary bypass procedures (eg, valve procedures) or for re-operation for coronary bypass more than 1 month after original operation	107
00563	Anesthesia for procedures on heart, pericardial sac, and great vessels of chest; with pump oxygenator with hypothermic circulatory arrest	105
01820	Anesthesia for all closed procedures on radius, ulna, wrist, or hand bones	102
00220	Anesthesia for intracranial procedures; cerebrospinal fluid shunting procedures	97
00930	Anesthesia for procedures on male genitalia (including open urethral procedures); orchiopexy, unilateral or bilateral	96
00100	Anesthesia for procedures on salivary glands, including biopsy	88
01270	Anesthesia for procedures involving arteries of upper leg, including bypass graft; not otherwise specified	85
01215	Anesthesia for open procedures involving hip joint; revision of total hip arthroplasty	84
01320	Anesthesia for all procedures on nerves, muscles, tendons, fascia, and bursae of knee and/or popliteal area	84
01924	Anesthesia for therapeutic interventional radiological procedures involving the arterial system; not otherwise specified	84
00144	Anesthesia for procedures on eye; corneal transplant	74
00802	Anesthesia for procedures on lower anterior abdominal wall; panniculectomy	74
01930	Anesthesia for therapeutic interventional radiological procedures involving the venous/lymphatic system (not to include access to the central circulation); not otherwise specified	74
00567	Anesthesia for direct coronary artery bypass grafting; with pump oxygenator	73
01120	Anesthesia for procedures on bony pelvis	71
00350	Anesthesia for procedures on major vessels of neck; not otherwise specified	67

© 2023 Lou SS et al. *JAMA Network Open*.

01965	Anesthesia for incomplete or missed abortion procedures	66
00750	Anesthesia for hernia repairs in upper abdomen; not otherwise specified	62
01730	Anesthesia for all closed procedures on humerus and elbow	61
00800	Anesthesia for procedures on lower anterior abdominal wall; not otherwise specified	60
00851	Anesthesia for intraperitoneal procedures in lower abdomen including laparoscopy; tubal ligation/transection	59
00700	Anesthesia for procedures on upper anterior abdominal wall; not otherwise specified	58
01716	Anesthesia for procedures on nerves, muscles, tendons, fascia, and bursae of upper arm and elbow; tenodesis, rupture of long tendon of biceps	56
00450	Anesthesia for procedures on clavicle and scapula; not otherwise specified	55
00794	Anesthesia for intraperitoneal procedures in upper abdomen including laparoscopy; pancreatectomy, partial or total (eg, Whipple procedure)	52
01112	Anesthesia for bone marrow aspiration and/or biopsy, anterior or posterior iliac crest	52
00792	Anesthesia for intraperitoneal procedures in upper abdomen including laparoscopy; partial hepatectomy or management of liver hemorrhage (excluding liver biopsy)	51
01916	Anesthesia for diagnostic arteriography/venography	51

© 2023 Lou SS et al. *JAMA Network Open*.
