# Supplementary material for: Anesthesia Clinical Workload Estimated From Electronic Health Record Documentation vs Billed Relative Value Units
Source: JAMA Netw Open. 2023 Aug 11;6(8):e2328514. doi: 10.1001/jamanetworkopen.2023.28514 (PMC10422189; doi:10.1001/jamanetworkopen.2023.28514)
Supplement: Supplement 2. — Data Sharing Statement [file jamanetwopen-e2328514-s002.pdf]

# Data Sharing Statement

Lou. Anesthesia Clinical Workload Estimated From Electronic Health Record Documentation vs Billed Relative Value Units. *JAMA Netw Open*. Published August 11, 2023. doi:10.1001/jamanetworkopen.2023.28514

**Data**
**Data available:** No

**Additional Information**
**Explanation for why data not available:** Deidentified data will be available on reasonable request.
